# Freeze-Derived Anisotropic Porous Microparticles for Engineered Mesenchymal Stem Cell Loading and Wound Healing

**DOI:** 10.34133/research.0668

**Published:** 2025-04-22

**Authors:** Rongwei Cai, Shuangshuang Miao, Xinyue Cao, Min Nie, Yuanjin Zhao

**Affiliations:** ^1^ Department of Rheumatology and Immunology, Nanjing Drum Tower Hospital, School of Biological Science and Medical Engineering, Southeast University, Nanjing 210096, China.; ^2^ Oujiang Laboratory (Zhejiang Lab for Regenerative Medicine, Vision, and Brain Health), Wenzhou Institute, University of Chinese Academy of Sciences, Wenzhou, Zhejiang 325001, China.

## Abstract

Hydrogel microparticles that can effectively deliver mesenchymal stem cells (MSCs) are expected to accelerate wound repair progress. Attempts in the area are focusing on improving the functions of the microparticles and MSCs to promote the therapeutic effect. Here, inspired by the topological morphology of ice branches, we propose novel freeze-derived anisotropic porous microparticles for hepatocyte growth factor (HGF)-overexpressing MSCs (MSCs^HGF^) loading and wound healing. The microparticles were fabricated by introducing microfluidic methacrylated gelatin pre-gel droplets into low-temperature silicone oil, followed by photo-cross-linking and freeze-drying processes. Drawing an advantage from the biocompatible chemical composition and the structured pore arrangement of the microparticles, MSCs^HGF^ can be efficiently encapsulated and released, maintaining continuous HGF secretion to enhance cell migration and support vascular regeneration. Leveraging these characteristics, we have shown that MSCs^HGF^-loaded porous microparticles could substantially promote angiogenesis, polarize macrophages toward the M2 phenotype, and reduce inflammation during the wound repair process, consequently enhancing skin wound repair efficiency. Thus, we believe that our MSCs^HGF^-integrated freeze-derived anisotropic porous microparticles hold promising prospects for clinical wound-healing applications.

## Introduction

The treatment of defective skin has attracted substantial attention in the medical field [1–3]. To date, numerous efforts have been devoted to facilitating the wound-healing process. These approaches to enhancing damaged tissue repair and regeneration encompass gene editing [4], regenerative medicine [5], and stem-cell-based therapies [6,7]. Mesenchymal stem cells (MSCs), which can transform into a multitude of cells, such as fibroblasts, myofibroblasts, and endothelial cells, are crucial in the healing process of wounds [8,9]. However, the challenge of poor MSC engraftment has hindered their effectiveness in treating tissue injuries across multiple models [10–12]. Fortunately, MSC-loaded hydrogel tissue-engineered scaffolds can improve cell viability and facilitate the wound-healing process [13,14]. Despite significant advances, existing hydrogel scaffolds exhibit relatively simple 3-dimensional structures and thus lack favorable topological features essential for promoting effective cellular adhesion and growth [15–17]. In addition, the constrained secretion of growth factors by conventional MSCs, coupled with limitations in structural design and cellular capacity, hinders their therapeutic efficacy and clinical applications [18–20].

**Fig. 1.**
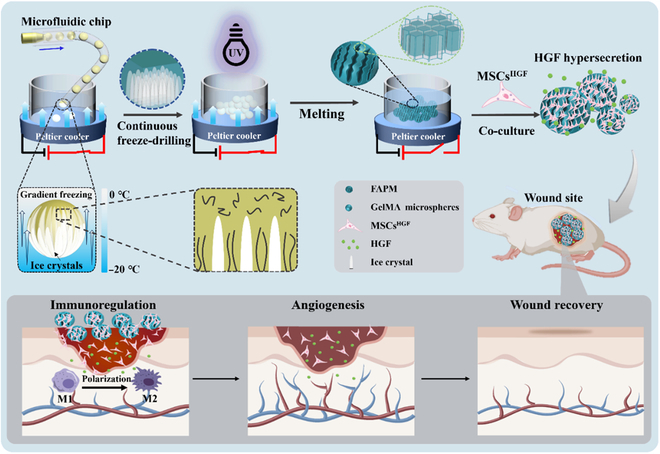
Scheme of the fabrication process and application. Schematic illustration of the fabrication of freeze-derived anisotropic porous microparticles (FAPMs). The hepatocyte-growth-factor-overexpressing mesenchymal stem cell (MSCs^HGF^)-loaded FAPMs are implanted into rats for wound healing. UV, ultraviolet; GelMA, methacrylated gelatin; HGF, hepatocyte growth factor.

Here, inspired by the topological morphology of ice branches [21–23], we propose novel freeze-derived anisotropic porous microparticles (FAPMs) for hepatocyte growth factor (HGF)-overexpressing MSCs (MSCs^HGF^) loading and wound healing, as schemed in Fig. 1. HGF is a type of multipotent growth factor with angiogenic, antifibrotic, and anti-inflammatory effects [24,25]. A wealth of research has shown that engineered MSCs can overexpress HGF and accelerate the wound-healing process [26–29]. In contrast, it has been demonstrated that by freeze-drilling and freeze-drying, desired porous microparticles can be obtained by replicating the microscale structures of ice crystals [30–32]. Although with much progress in freeze-casting hydrogel microparticles, most existing ice-templated porous microparticles are poorly designed due to the lack of control over the process of ice crystal formation [12,33]. Thus, if we can precisely control the development of ice crystals within porous microparticles, we are expected to obtain anisotropic porous microparticles. Meanwhile, engineered MSCs could be loaded by these porous microparticles with orientated pores. This system is expected to make great progress in the field of wound healing.

In this study, we fabricated the desired FAPMs through the combination of droplet microfluidics and ice-templating techniques for MSCs^HGF^ culture and wound-healing application, as schemed in Fig. 1. The microparticles were formed by introducing microfluidic methacrylated gelatin (GelMA) pre-gel droplets into low-temperature silicone oil placed on a Peltier cooler, where the sharp temperature gradient caused the GelMA droplets to freeze from the bottom upward, leading to the compression and accumulation of GelMA molecules by the ice crystals. Thus, by photo-cross-linking the GelMA and removing ice crystals by freeze-drying, porous microparticles with a microscale ice-branch-like structure can be obtained. Due to this specific structure and the inherent cell-adhesive properties of GelMA, MSCs^HGF^ could easily proliferate and migrate within the porous microparticle scaffolds. As their constantly secreted HGF could promote cell migration and vascular regeneration, the MSCs^HGF^-loaded porous microparticles were demonstrated to effectively promote angiogenesis, polarize macrophages toward the M2 phenotype, and reduce inflammation during the wound repair process, consequently enhancing skin wound repair efficiency. These results indicate that FAPMs integrated with MSCs^HGF^ hold promising prospects for tissue regeneration and have great potential in medical applications.

## Results

**Fig. 2.**
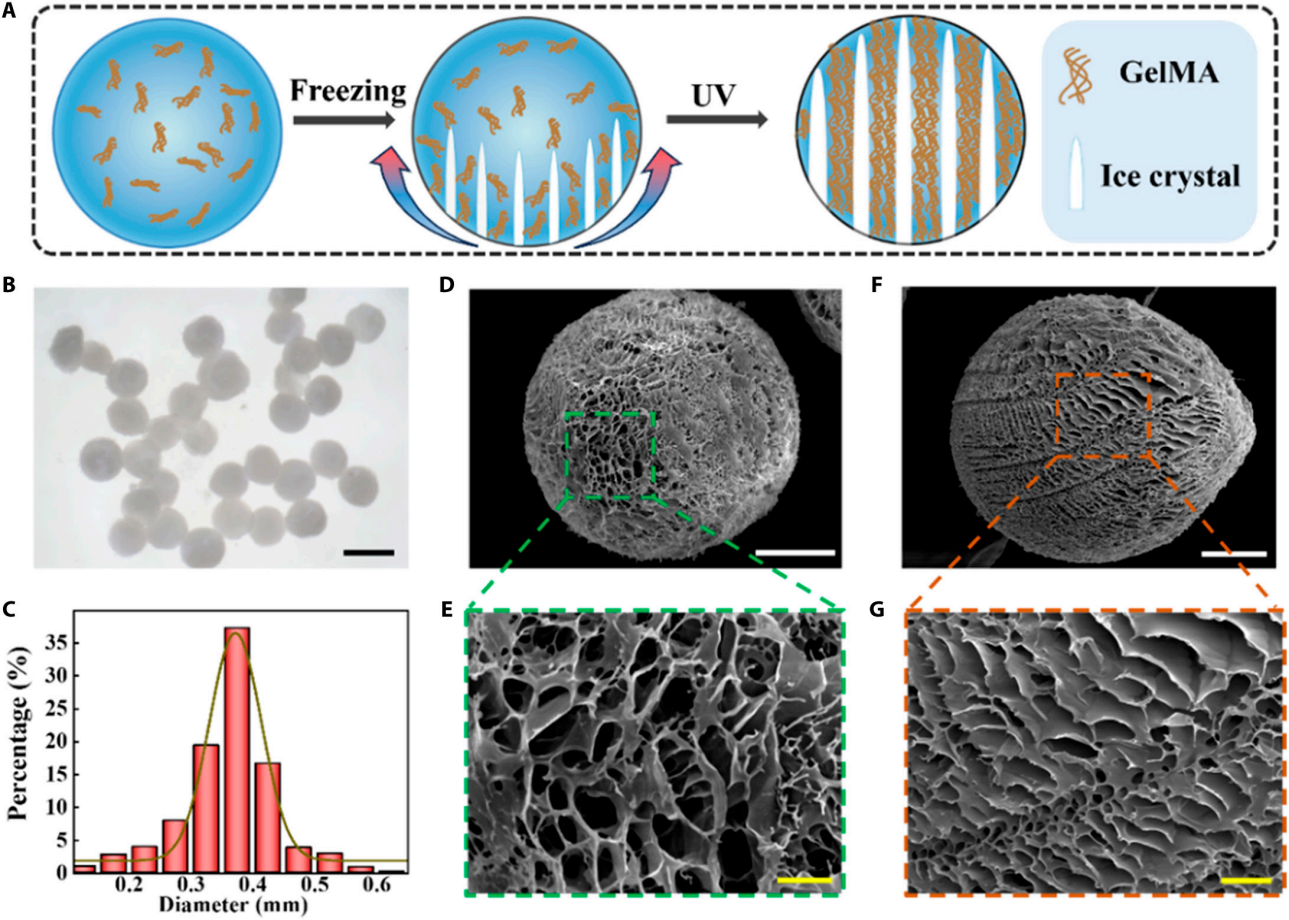
Fabrication and characterization of FAPMs. (A) The formation process of freeze-derived microparticles. (B) Microscopic image of the FAPMs. The scale bar is 500 μm. (C) The size distribution of the resultant microdroplets. (D and E) Scanning electron microscopic (SEM) images of normal freeze-dried microparticles. (F and G) SEM images of FAPMs. The scale bar is 100 μm in (D) and (F). The scale bar is 20 μm in (E) and (G).

In a typical experiment, we generated FAPMs with an anisotropic microstructure by microfluidics and directional freeze-casting (Fig. 2A). Microfluidics, an effective instrument for accurately manipulating fluids and their interface, enabled the creation of monodisperse FAPMs by accurately dispersing aqueous GelMA precursors within the outer silicone oil phase. Figure S1a illustrates the detailed formation process, where the 2 phases flowing together in a microfluidic chip resulted in the dispersion of the GelMA precursor into microdroplets. Notably, the diameter of the solidified microdroplets demonstrated a direct positive correlation with the inner-phase flow rate and an inverse correlation with the outer-phase flow rate (Fig. S1b and c). By adjusting the flow velocities of the GelMA precursor and silicone oil, the size of these microparticles can be accordingly regulated. After the microfluidic fabrication, these GelMA microdroplets were collected and placed in a steady gradient-freezing environment, which was constructed by placing a mold containing methyl silicone oil on a Peltier cooler for approximately 10 min (Fig. S2). Because of the vertical temperature gradient, ice nucleation occurred in the colder region of the microdroplet, and ice crystals extended from the ice nucleation site toward the warmer area of the microdroplet. After gradient directional freezing for 20 min (Fig. S3), the microdroplets were solidified by ultraviolet (UV) photo-cross-linking. Subsequently, after removing silicone oil, the ice-structured microparticles were stored in a liquid nitrogen environment to further immobilize the aligned structures. Finally, FAPMs were obtained after vacuum freeze-drying (Fig. 2B). The collected microparticles exhibited a coefficient of variation of less than 5% (Fig. 2C), attesting to the exceptional monodispersity achieved through microfluidics. Notably, this size uniformity ensured the stability of the physicochemical properties of the resultant FAPMs.

Macroscopic analysis revealed distinct morphological disparities between FAPMs and traditional freeze-dried microparticles. Specifically, FAPMs maintained an opaque white morphology with preserved structural integrity, whereas conventional freeze-dried microparticles exhibited a translucent appearance indicative of homogeneous network formation (Fig. S4). The scanning electron microscopic images further validate the effect of the gradient temperature field on the final morphology of microparticles. Notably, the traditional freeze-dried microparticles exhibited a randomly oriented pore morphology (Fig. 2D), while the microparticles produced by the temperature-gradient-freezing process had anisotropic major-sized pores and branched pore morphology (Fig. 2F). Additionally, the microparticles directly normally freeze-dried after UV photopolymerization at room temperature showed circular pores with a size of about 20 to 40 μm (Fig. 2E). In contrast, the formed FAPMs displayed anisotropic pores with a size of 40 to 80 μm (Fig. 2G). These results indicate the important effect of temperature gradient freezing on pore formation. Given that, we further explored how gradient-freezing temperatures influenced the morphology of the microparticles. As shown in Fig. S5, FAPMs-R, FAPMs-10, and FAPMs-20 refer to the frozen temperatures of microparticles in the first stage of the cooling process. FAPMs-R were the microparticles prepared at room temperature, while FAPMs-10 and FAPMs-20 were microparticles prepared at −10 and −20 °C, respectively. It was evident that increasing the temperature gradient would lead to more oriented pores.

**Fig. 3.**
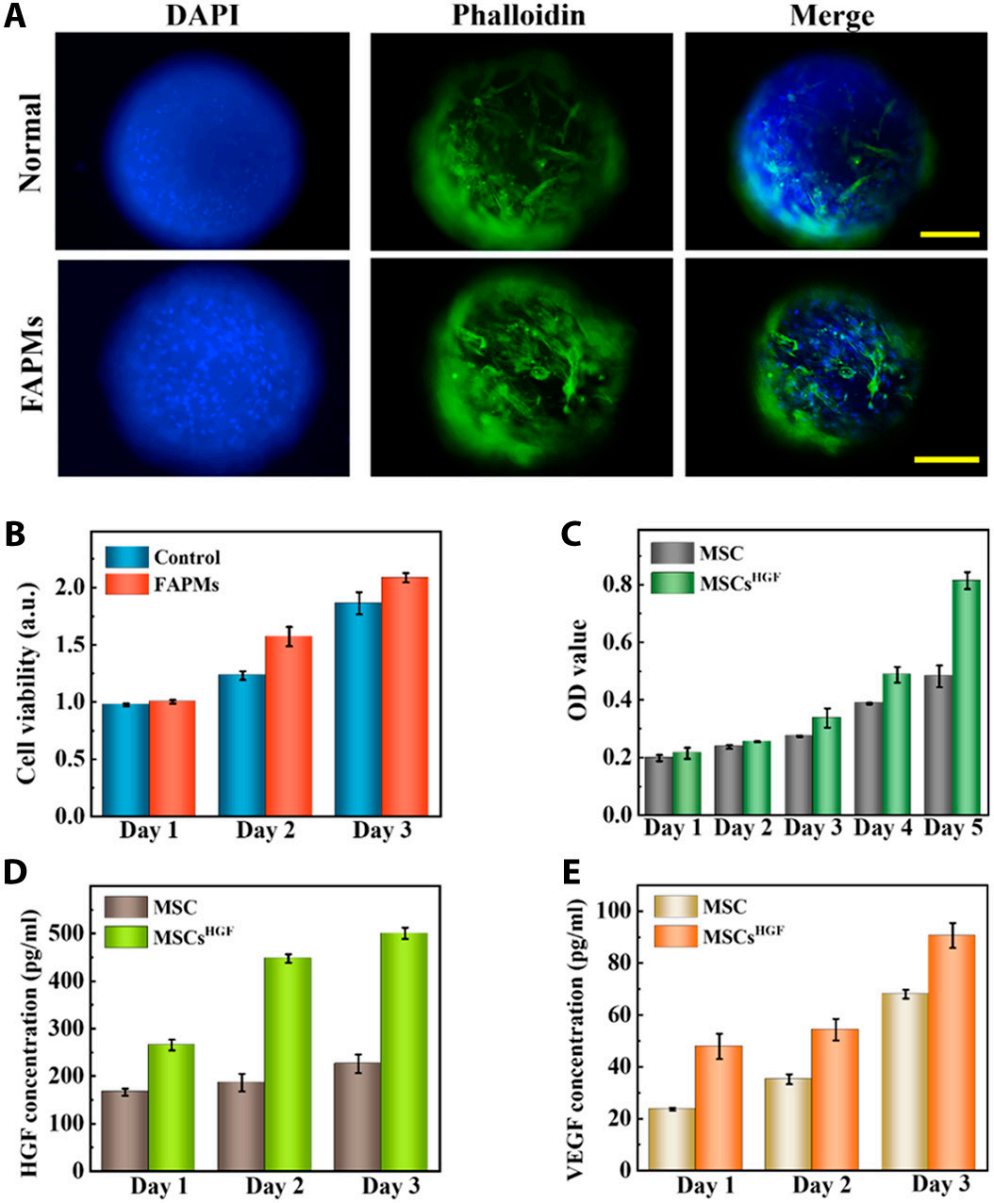
Verification of the load capacity of FAPMs on functional stem cells. (A) 4′,6-Diamidino-2-phenylindole (DAPI)/phalloidin staining of mesenchymal stem cells (MSCs) cultured on FAPMs; the scale bar is 200 μm. (B) The quantitative analysis of the MTT (3-(4,5-dimethylthiazol-2-yl)-2,5-diphenyltetrazolium bromide) results of FAPMs. (C) Cell proliferation. The quantitative analysis of the MSCs and MSCs^HGF^ was performed using Cell Counting Kit-8 (CCK-8). (D) The concentration of HGF in the culture supernatants of transfected/untransfected cells on days 1, 2, and 3 was detected by enzyme-linked immunosorbent assay (ELISA). (E) The concentration of vascular endothelial growth factor (VEGF) in the culture supernatants of transfected/untransfected cells on days 1, 2, and 3 was detected by ELISA. OD, optical density.

The FAPM scaffolds exhibited an excellent load capacity for functional stem cells. The capacity to facilitate initial cell adhesion and subsequent proliferation was a crucial characteristic of scaffolds used in wound healing. In this work, the microspheres’ biocompatibility and its effect on cell adhesion and proliferation were investigated. To investigate FAPMs’ biocompatibility, we co-cultured FAPMs and NIH-3T3 cells, using blank culture dishes as the control. As illustrated in Fig. S6, the fluorescence images of the 2 groups visually demonstrate the 3-d growth of NIH-3T3 cells. NIH-3T3 cultured with FAPMs proliferated well, showing no significant difference from the cells in the control group. The cell activity in the FAPM group also showed an ideal state on the third day (Fig. 3B). Additionally, we performed hemolysis experiments to further illustrate FAPMs’ blood compatibility. The absence of hemolysis in both the FAPMs and negative control groups is shown in Fig. S7. To examine MSCs’ spreading and adhering, fluorescence images were captured showing F-actin and nucleus staining after culturing for 3 d. The results were observed by a fluorescence microscope. It could be seen that MSCs could be uniformly and massively loaded on the FAPMs (Fig. 3A). Figure S8 also successfully shows that MSCs could proliferate on FAPMs, and more MSCs grew on FAPMs than on normal freeze-dried microparticles. This might be attributed to the excellent porosity and larger pore size of FAPMs.

Engineered MSCs have better cell viability and the ability to secrete HGF. Existing studies have proved that external HGF could promote healing of skin incisions [34,35]. However, using HGF solely has hardly sustained therapeutic power. Considering this disadvantage, lentivirus transfection was used to overexpress HGF in MSCs to repair wounds. Cell viability was crucial for FAPMs. We cultured MSCs and MSCs^HGF^ for 5 d and used Cell Counting Kit-8 (CCK-8) to demonstrate the viability changes of these 2 groups. As shown in Fig. 3C, MSCs^HGF^ showed better cellular activity over time, which facilitated cell adhesion and proliferation on the microparticles. Moreover, enzyme-linked immunosorbent assay (ELISA) analysis revealed that the content of HGF in the cell suspension from the transfected groups was elevated in comparison with that in the nontransfected group (Fig. 3D). The degree of HGF accumulation in cell suspension increased gradually with the extension of culture time. It has been demonstrated that HGF-modified MSCs could significantly amplify paracrine effects, which is a key mechanism in promoting the wound-healing process. Notably, vascular endothelial growth factor (VEGF) is a classic growth factor in the paracrine effect. To further investigate whether MSCs^HGF^ could enhance the paracrine impact of cells, an ELISA kit was also employed to measure the content of VEGF in the cell suspension. It was shown that the content of VEGF in the cell suspension of transfected groups was higher compared to that in the control group (Fig. 3E), confirming that MSCs^HGF^ has exhibited an enhanced paracrine effect.

**Fig. 4.**
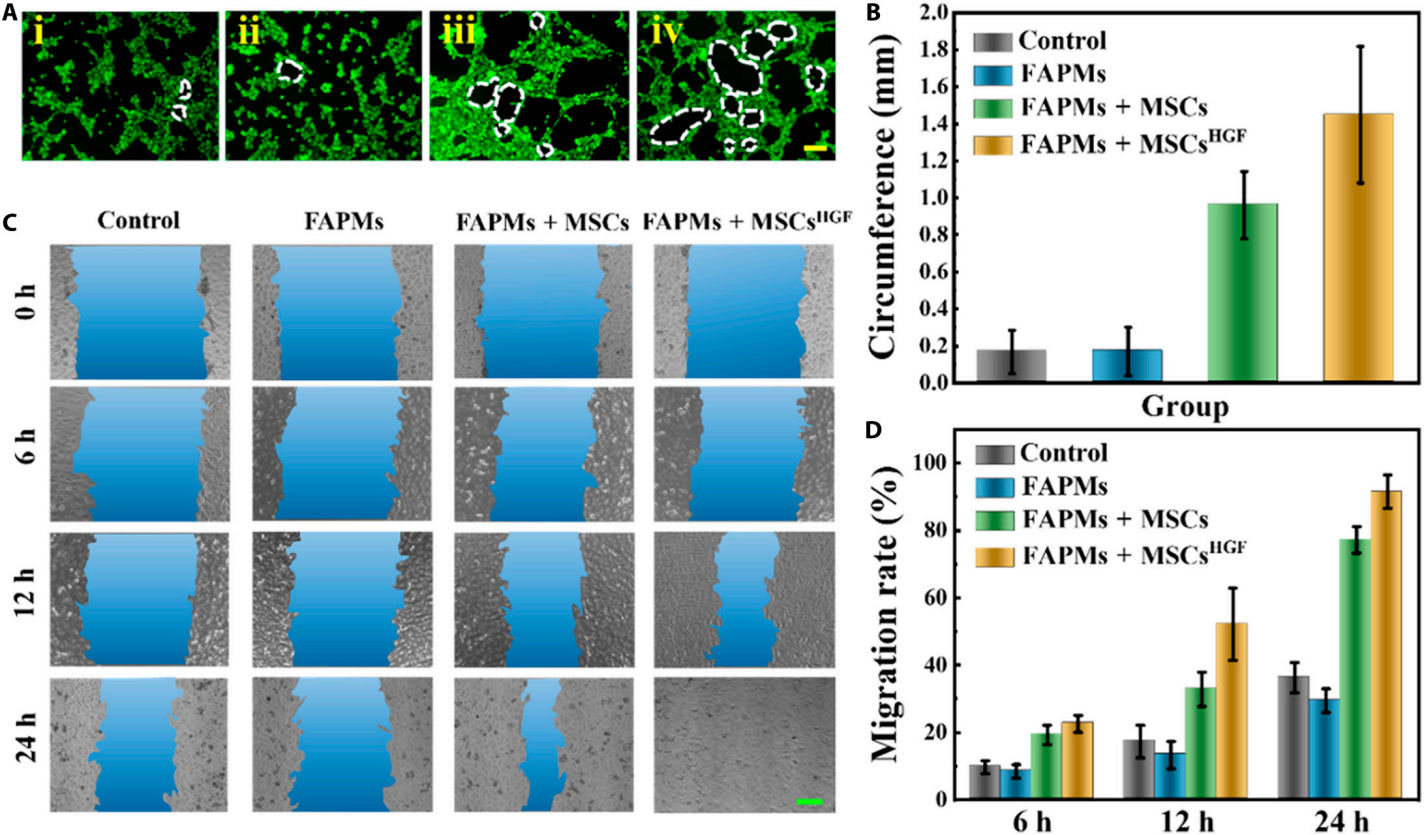
Biological functions of FAPMs loaded with MSCs^HGF^. (A) Fluorescence photographs of tube formation experiments in Matrigel. (i) Human umbilical vein endothelial cells (HUVECs) cultured in Dulbecco’s modified Eagle medium (DMEM), the leaching solution of FAPMs aged for 24 h (ii), the leaching solution of FAPMs loaded with MSCs aged for 24 h (iii), and the leaching solution of FAPMs loaded with MSCs^HGF^ aged for 24 h (iv). The scale bar is 100 μm. (B) Quantitative analysis of tube formation (*n* = 3). (C) Optical image of scratch assay of NIH-3T3 cells. The scale bar is 250 μm. (D) Quantitative analysis of cell migration (*n* = 3).

To test the biological function of MSCs^HGF^-loaded FAPMs, angiogenesis and cell scratching experiments were performed. HGF is essential for promoting vascularization and cell migration throughout the wound-healing process. To investigate if engineered MSCs^HGF^ possessed the same effective and targeted angiogenic capabilities as pure HGF, a tube formation assay was conducted in vitro. The experiment involved 3 treatment groups and a control group, with human umbilical vein endothelial cells cultured in all groups. After 12 h of culture, the FAPMs + MSCs^HGF^ group notably accelerated the formation of vascular cell networks (Fig. 4A). Furthermore, tube length analysis revealed a stronger angiogenesis-promoting effect in the FAPMs + MSCs^HGF^ group. (Fig. 4B). This could be attributed to the fact that MSCs^HGF^ secrete higher levels of HGF compared to conventional MSCs. Furthermore, the modification of MSCs enhanced the paracrine secretion of various growth factors, including VEGF, which subsequently facilitated the process of angiogenesis. Moreover, fibroblast migration plays an essential role in wound healing. In the scratch test, NIH-3T3 cells from the MSCs^HGF^-conditioned medium showed a significantly higher migration rate compared to the nontransfected cells, demonstrating the positive impact of the excessive secretion of HGF and VEGF by MSCs^HGF^ (Fig. 4C and D). Benefiting from the enhanced angiogenesis capability and the ideal ability to promote cell migration, FAPMs loaded with MSCs^HGF^ would have significant potential for future applications in wound healing.

**Fig. 5.**
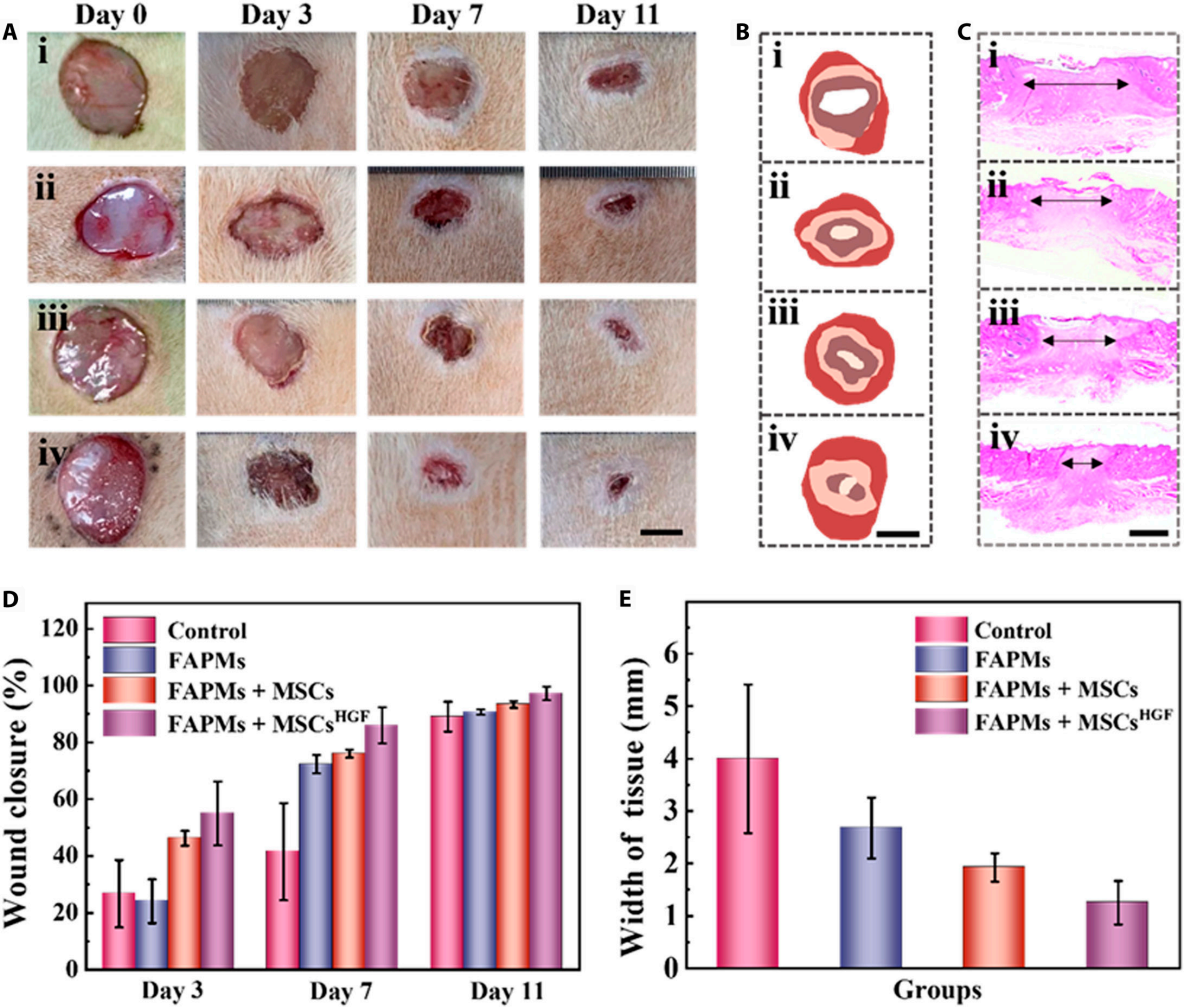
Wound closure process. (A and B) Images of the skin wound. The scale bars in (A) and (B) are 5 mm. (C) Images of hematoxylin and eosin (H&E) staining of wounds. The scale bar is 1 mm. (D) The statistical analysis of the wound closure situation (*n* = 3). (E) Quantitative measurement of granulation tissue width (*n* = 3). In (A) to (C), (i) is treated with phosphate-buffered saline (PBS) (control group), (ii) is treated with FAPMs, (iii) is treated with FAPMs + MSCs, and (iv) is treated with FAPMs + MSCs^HGF^.

To evaluate the therapeutic effects of FAPMs loaded with MSCs^HGF^, rat models of full-thickness skin defects were established and received different treatments. Sprague–Dawley rats were assigned to 4 different groups: FAPMs, FAPMs + MSCs, FAPMs + MSCs^HGF^, and a control group with phosphate-buffered saline (PBS). To directly show the healing status, the wound areas were photographed on days 0, 3, 7, and 11. It was observed that across all the groups, the rats in the FAPMs + MSCs^HGF^ group exhibited the most rapid and noticeable reduction in wound size, achieving a wound closure rate of 97.21% ± 2.39% by day 11 (Fig. 5A, B, and D). Moreover, no substantial difference in healing rate was found between the FAPMs and the baseline group, suggesting that the cytokines released from the FAPMs contributed significantly. The MSC group showed less effective wound recovery compared to the FAPMs + MSCs^HGF^ group, which might benefit from the promoted cell migration and proliferation condition because of the oversecreted HGF. To gain further insight into the in vivo effects of FAPMs loaded with MSCs^HGF^, histopathological analysis was performed. For this reason, hematoxylin and eosin staining of the rats’ wound beds on day 11 was conducted (Fig. 5C). The images revealed that compared to the other groups, the rats treated with FAPMs loaded with MSCs^HGF^ exhibited the least expansive granulation tissue, suggesting superior tissue regeneration and wound repair (Fig. 5E). Based on these results, it is believed that the FAPMs loaded with MSCs^HGF^ would have potential practical application value in the wound management field.

**Fig. 6.**
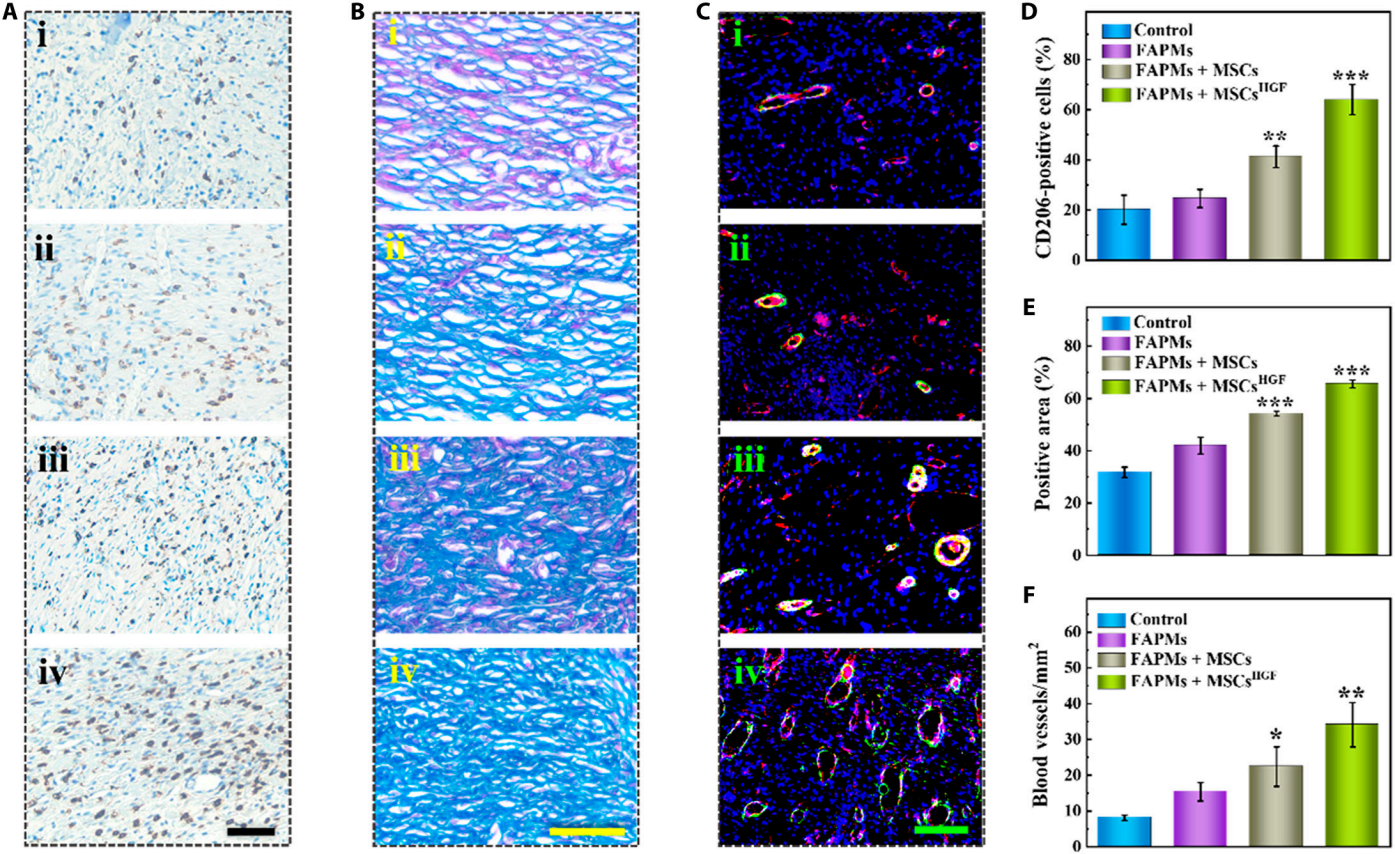
Immunostaining analysis of tissues. (A) Immunostaining of CD206 in 4 experimental groups. (B) Masson’s trichrome staining of 4 experimental groups. (C) CD31 and α-smooth muscle actin (α-SMA) immunofluorescent staining of 4 experimental groups. (i) is the control group, (ii) is the FAPM group, (iii) is the FAPMs + MSCs group, and (iv) is the FAPMs + MSCs^HGF^ group. Statistical analysis of (D) CD206 and (E) collagen deposition and (F) quantitative analysis of the blood vessel density in 4 groups. Scale bars are all 200 μm. A Student *t* test was performed to compare the control group and experimental groups. *P < 0.05; **P < 0.01; ***P < 0.001. Data are presented as mean ± SD.

Furthermore, to examine the inflammation and angiogenesis at the wound site, immunohistochemical (IHC) staining was performed to estimate the expression of critical inflammatory biomarkers in the wound bed. During the initial stage of wound healing, interleukin-6 and tumor necrosis factor-α were frequently used as the standard inflammatory markers, with their expression being assessed to determine the level of wound infection. Their expression was analyzed through IHC staining on day 11. The staining data revealed the FAPMs + MSCs^HGF^ group had the least secretion of interleukin-6 and tumor necrosis factor-α compared to the other 3 experimental groups, indicating the mildest inflammatory response (Figs. S9 and S11). Inflammatory response, particularly the shift of macrophages to the M2 phenotype, plays a crucial role in tissue regeneration. MSCs have been shown to promote this polarization, and HGF further enhances this effect by converting M1 macrophages to M2. HGF synergistically potentiates this immunomodulatory cascade through mesenchymal–epithelial transition factor (MET) receptor-mediated mechanism receptor-mediated mechanisms [36,37]. To further classify the macrophage subtypes, we utilized IHC staining with the M2-specific marker CD206 to determine the count of M2 macrophages (Fig. 6A and D). All experimental groups exhibited a higher number of M2 macrophages in the wound beds than the control group, and the FAPMs + MSCs^HGF^ group displayed the highest concentration of M2 macrophages. In contrast, no considerable difference in the number of M2 phenotype macrophages was identified between the FAPM group and the control group. In contrast, the M1 macrophage number exhibited a contrary corresponding relation with the 4 groups (Fig. S11). These results indicate that the FAPMs loaded with MSCs^HGF^ were capable of promoting macrophage polarization toward the M2 phenotype, thus aiding tissue restoration.

Additionally, in the final stage of wound healing, collagen deposition within the wound bed functions as a crucial indicator that could provide insight into the status of tissue remodeling condition. Masson staining was performed on skin specimens to examine the arrangement of collagen fibers within the wound sites. The results revealed that the FAPMs + MSCs^HGF^ group exhibited more compact collagen accumulation and a more organized fiber structure compared to the other 3 groups (Fig. 6B and E). Angiogenesis is a key factor in evaluating the remodeling condition of the tissue. It is noted that CD31 and α-smooth muscle actin (α-SMA) are common markers associated with vascular smooth muscle and endothelial cells. Therefore, immunofluorescence staining was utilized to investigate the expression of CD31 and α-SMA at the wound site to assess angiogenesis at the wound. However, due to the characteristics of MSCs in stimulating angiogenesis, the FAPMs + MSCs and FAPMs + MSCs^HGF^ groups showed an increased number of newly formed blood vessels in the wound section when contrasted with the control group and FAPM group. Due to the role of HGF in promoting angiogenesis, the FAPMs + MSCs^HGF^ group exhibited a higher concentration of new blood vessels at the wound site (Fig. 6C). Furthermore, the quantitative results of these biomarkers verified the analysis from these staining images (Fig. 6F). These characteristics indicate that FAPMs loaded with FAPMs + MSCs^HGF^ hold significant promise for enhancing wound healing.

## Discussion

We have developed an innovative system utilizing FAPMs for the efficient delivery of MSCs^HGF^ to enhance wound healing. These microparticles with unique anisotropic porous architecture allow the nondestructive capture and abundant delivery of MSCs^HGF^. Benefiting from the biocompatible chemical composition and aligned porous structure of the microparticles, MSCs^HGF^ can proliferate and migrate within the scaffolds while continuously secreting HGF. This constant secretion promotes cell migration and vascular regeneration, effectively enhancing angiogenesis, polarizing macrophages toward the M2 phenotype, and reducing inflammation during the wound repair process. Consequently, MSCs^HGF^-loaded porous microparticles significantly improve skin wound repair efficiency. It has been demonstrated that FAPMs integrated with MSCs^HGF^ can promote vascularization, tissue regeneration, and collagen deposition in the wound bed. Thus, such MSCs^HGF^-loaded FAPMs showed significant potential for great application prospects in the wound-healing field.

## Materials and Methods

### Preparation of the GelMA pre-gel suspension

GelMA and H_2_O were combined in a 1:1 w/v ratio of 1:1, and then 2-hydroxy-2-methylpropiophenone (2% v/v) was added into the mixture. After thoroughly blending them, a GelMA pre-gel suspension was obtained.

### Microfluidics

The microfluidic setup was configured as a coflow arrangement. GelMA microparticles were injected through an inner capillary, while methyl silicone oil entered via an outer capillary. Shear and surface tension forces caused the inner phase to break up into uniform microdroplets. To regulate the microparticles’ size, the flow rates of both phases were controlled using a syringe pump (Longer Pump LSP01).

### Fabrication of FAPMs

FAPMs were fabricated using a freeze-photopolymerization technique. A small mold filled with methyl silicone oil was positioned on a Peltier cooler, separated by a thin ethanol liquid layer. The GelMA pre-gel suspension was introduced slowly and evenly through the microfluidic system. The pre-placed methyl silicon oil provided a gradient-freezing environment from the bottom up, so the suspension’s water content was gradually frozen. After approximately 30 s of UV exposure, the ice crystal structure was replicated. Finally, removing the ice template at room temperature led to the formation of the FAPMs with randomly oriented pore structures.

### Biocompatibility test of FAPMs

Initially, FAPMs underwent a sterilization process employing UV light for 24 h. Subsequently, these sterilized FAPMs were carefully placed into 48-well culture plates. Within this experimental setup, NIH-3T3 cells were introduced into the culture plates to serve as the control group, while another set of cells were cultured on FAPMs, constituting the experimental groups. A mixture of 50 μl of MTT solution and 500 μl of culture solution was prepared and applied to the cultured cells, allowing a 4-h incubation period. Subsequently, the resulting crystals were dissolved in the 600 μl of dimethyl sulfoxide. Finally, the optical absorbance of the resulting solution was then measured.

### In vitro cell culture

MSCs were isolated from human umbilical cord tissues. The green fluorescent protein-tagged hepatocyte growth factor (GFP-HGF) lentivirus was manufactured by Nanjing Zebrafish Biotech Co., Ltd. MSCs^HGF^ were prepared through lentivirus-mediated gene transfection and screened using puromycin. The MSCs and MSCs^HGF^ were cultured on FAPMs. FAPMs underwent sterilization via UV irradiation for 1 d and were rinsed with PBS 3 times. The MSCs were maintained in a 5% CO_2_ atmosphere at 37 °C. Once the cells’ area reached 90%, EDTA was applied to detach the cells from the flasks, and they were subsequently transferred to a T25 flask for continued culture.

### Matrigel tube formation assay

Matrigel was evenly applied to the surfaces of 48-well culture plates, creating a symmetrical coating. Subsequently, human umbilical vein endothelial cells, suspended in solution, were carefully cultured into the coated wells with a concentration of 10^4^ cells per well. The culture plates were then categorized into 4 distinct groups based on the composition of the medium employed: the control group, consisting of blank culture medium; the FAPM group, featuring medium supplemented with FAPMs; the MSC group, containing medium enriched with extracellular solution co-cultured with FAPMs and MSCs; and finally, the MSCs^HGF^ group, wherein the extracellular solution was co-cultured with FAPMs and MSCs^HGF^. Each group comprised 3 replicates. After incubation for 6 h, fluorescent staining was undertaken to analyze the formation of tubes on the culture plates.

### Scratch test

NIH-3T3 cells were plated in an experimental plate and maintained until they reached over 90% cell coverage. After 6 h without nutrients, the cell monolayer was gently scraped with a pipette tip. PBS was used to remove the detached cells. There were 4 groups: the control group (treated with serum-free Dulbecco’s modified Eagle medium [DMEM]), the FAPM group (treated with 24-h-aged serum-free DMEM leachate from FAPMs), the MSC group (treated with 48-h-aged serum-free DMEM leachate from MSC-loaded FAPMs), and the MSCs^HGF^ group (treated with 48-h-aged serum-free DMEM leachate from MSCs^HGF^-loaded FAPMs), with 3 parallel trials per group. Photographs were taken at 0, 6, 12, and 24 h following the scraping procedure.

### Detection of HGF and VEGF content in the wounds by ELISA

The MSCs and MSCs^HGF^ were cultured in a T25 flask. When the area of cells reached 80%, EDTA was used to digest the cells from flasks and a cell counting plate was used to confirm the density of cells. On days 1, 2, and 3, the suspension of both cell types was obtained. The content of HGF and VEGF was detected by ELISA following the introduction of the HGF and VEGF ELISA kit.

### Wound-healing study in vivo

Rats weighing between 190 and 210 g were organized into 4 groups: the control group (PBS), FAPMs, FAPMs loaded with MSCs (FAPMs + MSC), and FAPMs loaded with MSCs^HGF^ (FAPMs + MSCs^HGF^ ). After anesthesia and shaving, a circular wound approximately 1 to 1.5 cm in diameter was made on each rat’s back. Subsequently, 200 μl of PBS, FAPMs, FAPMs loaded with MSCs, and FAPMs loaded with MSCs^HGF^ was administered around the wound area in their respective groups. The wound-healing process was monitored through regular photographs, and regenerated tissue was collected on day 11 for further analysis.

### Histological, IHC, Masson, and immunofluorescence staining

All collected tissues were fixed in a 4% (v/v) paraformaldehyde solution for 24 h, followed by a stepwise dehydration process using ethanol solutions. The tissues were initially immersed in different concentrations of ethanol for different periods of time to achieve the final process of complete dehydration. Following dehydration, the tissues were infiltrated with paraffin before being sliced into 5-μm layers. Histological analysis was performed utilizing either hematoxylin and eosin or Masson staining protocols. IHC staining was carried out to evaluate the inflammation level, while double immunofluorescence staining with CD31 and α-SMA antibodies was employed to observe the formation of new blood vessels. Additionally, inducible nitric oxide synthase staining was employed to assess the quantity of M1 macrophages, while CD206 staining was utilized to quantify M2 macrophages.

### Statistical evaluation

The data were normalized using the control group as a reference, and the results are shown as mean ± standard deviation (SD). The number of samples (*n*) for each analysis is specified in each figure’s caption. A Student *t* test was applied to assess significant differences. *P < 0.05; **P < 0.01; ***P < 0.001. Statistical analysis was carried out using Origin and ImageJ.

## Data Availability

All data are available online on the *Research* website.
