# Supplementary material for: Freeze-Derived Anisotropic Porous Microparticles for Engineered Mesenchymal Stem Cell Loading and Wound Healing
Source: Research (Wash D C). 2025 Apr 22;8:0668. doi: 10.34133/research.0668 (PMC12012297; doi:10.34133/research.0668)
Supplement: Supplementary 1 — Figs. S1 to S11 [file research.0668.f1.zip › Supplementary Information.docx]

Supplementary Materials

**
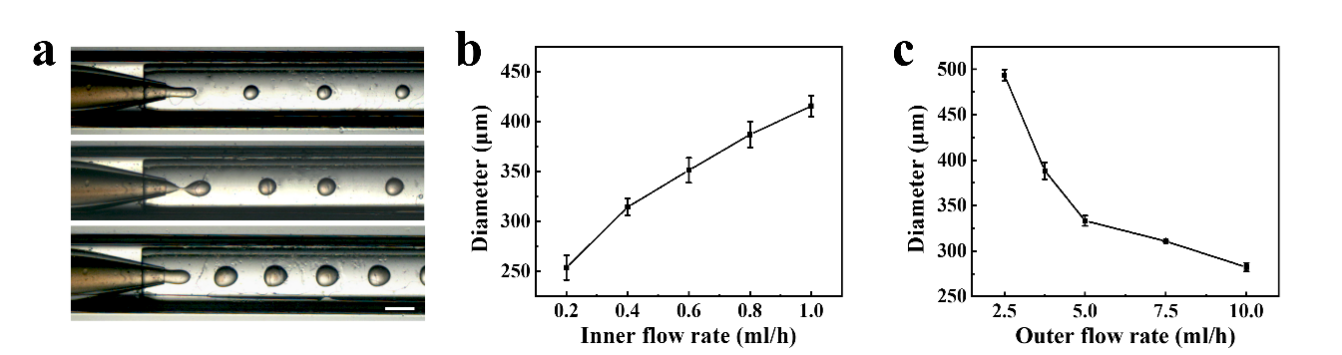
**

**Figure. S1 | Microfluidic control of microsphere size. (a)** The fabrication process of the GelMA droplet within the microfluidic channel. The scale bar is 500 μm. **(b)** The plot of microparticle diameter versus internal phase flow rate for a fixed external phase flow rate (n=3). **(c)** Plot of microparticle diameter versus external phase flow rate for a fixed internal phase flow rate (n=3).


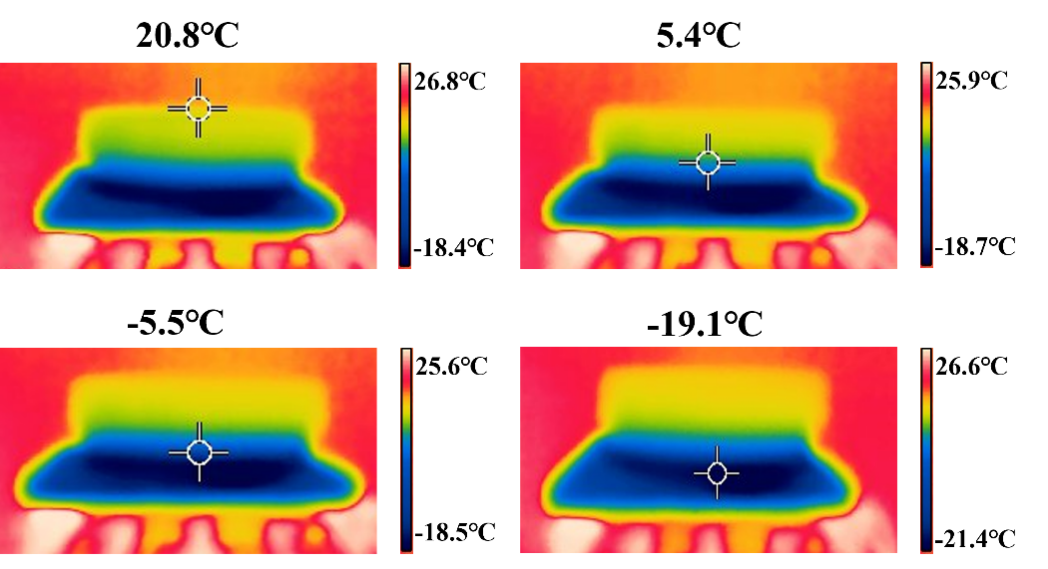


**Figure. S2 |Thermal image of gradient freezing field.**


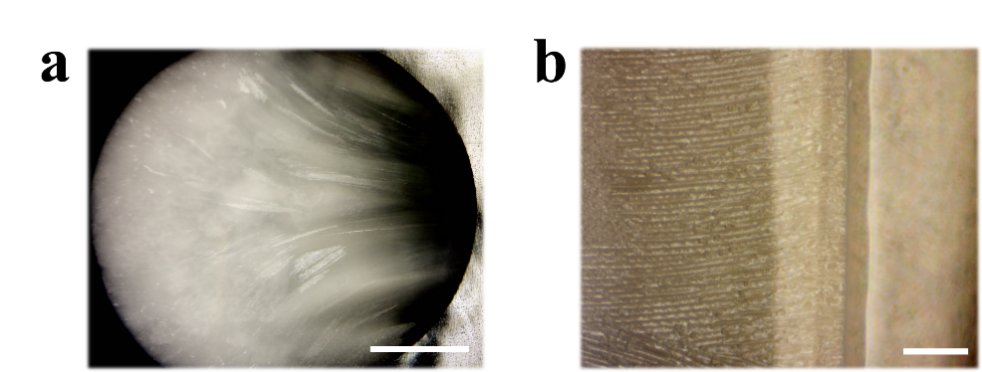


**Figure. S3 |** **Gradient freezing image of droplets and films of GelMA. (a)** Droplet. The scale bar is 500 μm. **(b)** Film. The scale bar is 100 μm.


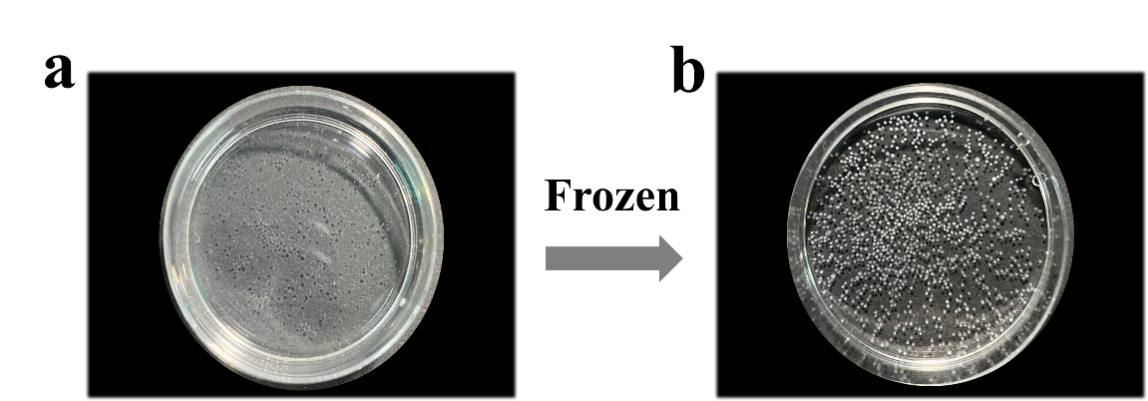


**Figure. S4 |** **Macroscopic observation images of conventional freeze-drying microparticles** **and FAPMs. (a)** conventional freeze-drying microparticles. **(b)** FAPMs.


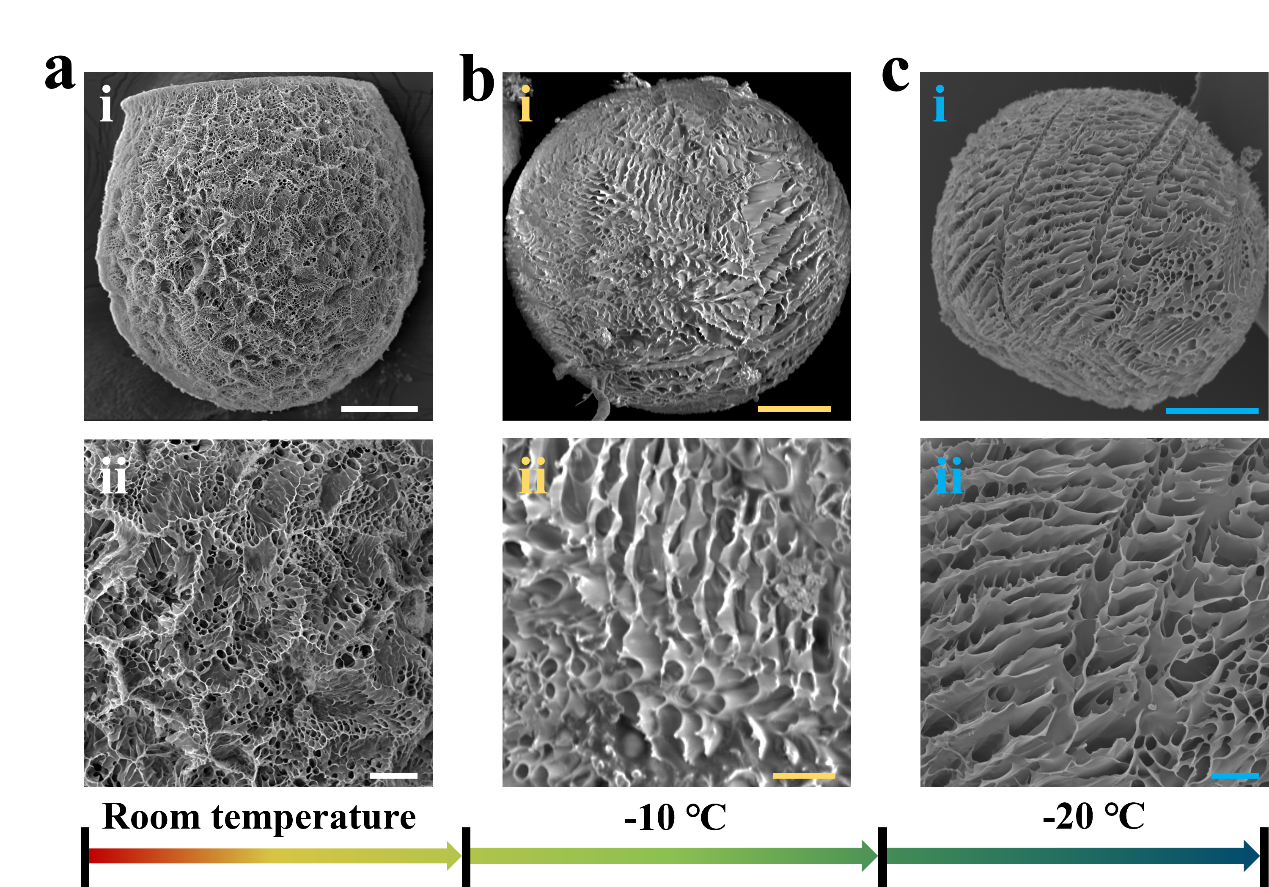


**Figure. S5 | SEM images of FAPMs at different temperature gradients. (a)** is the room temperature group, **(b)** is the -10℃ group, and **(c)** is the -20℃ group. (i) is the overall picture of the FAPMs, and (ii) is the details. The scale bars in (i) are all 100 μm and in (ii) are all 20 μm.


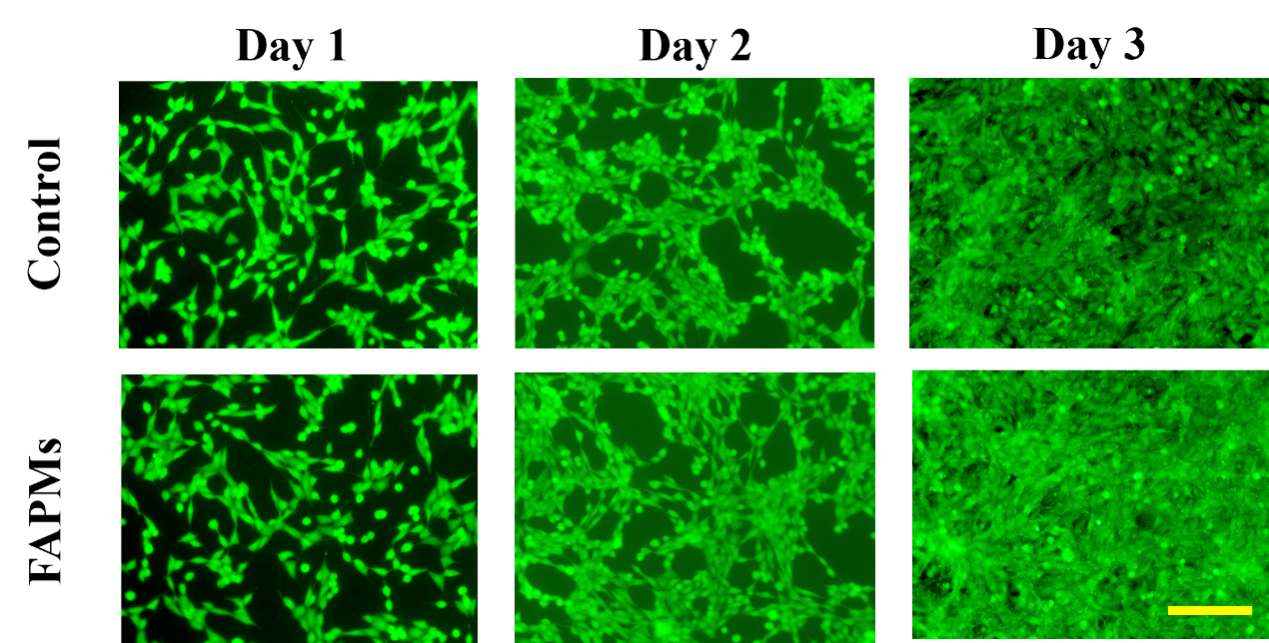


**Figure. S6 | Fluorescence microscopy results of biocompatibility.** The scale bar is 200 μm.

**
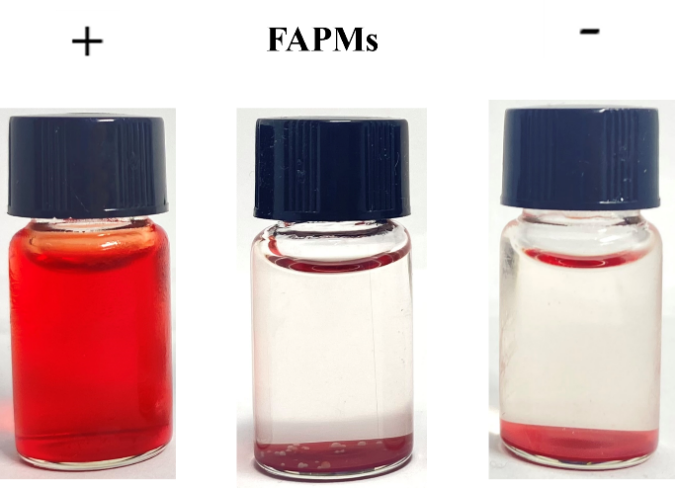
**

**Figure. S7 |** **Optical images of the result of the hemolysis test.**

**
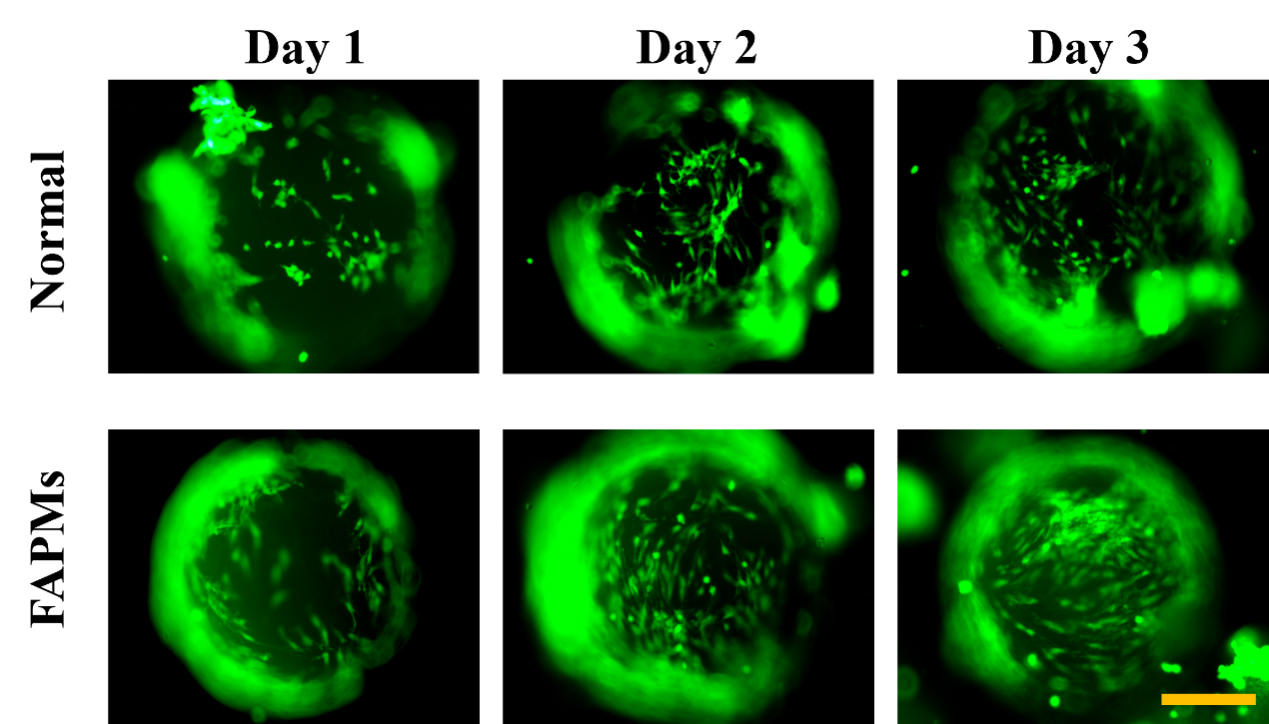
**

**Figure. S8 | Fluorescence microscope images of FAPMs and ordinary microparticles co-cultured with MSCs cells for 3 days.** The scale bar is 200 μm.


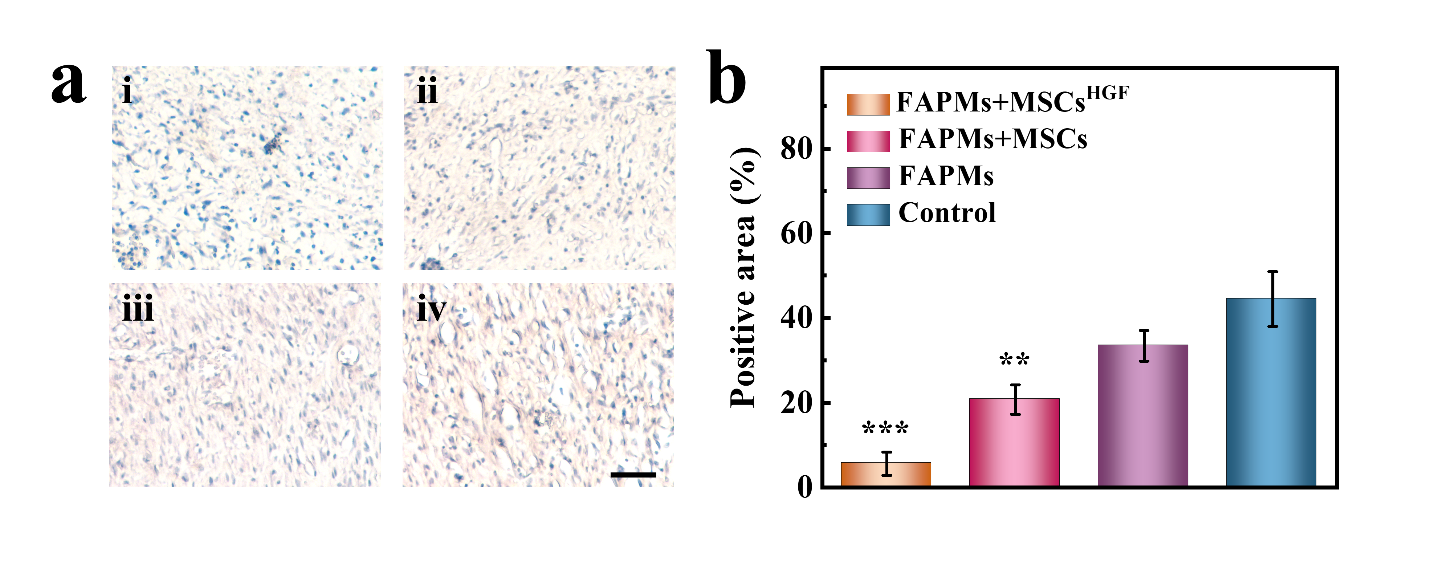


**Figure. S9 | Immunostaining of IL-6 of granulation tissue in different groups.** **(a)** Microscope photographs. **(b)** Statistical analysis (n=3). i) the FAPMs+MSCs^HGF^ group, ii) the FAPMs+MSC group, iii) the FAPMs groups, and iv) the control group. The scale bar is 200 μm. A Student's *t*-test was performed to compare the experimental groups and experimental groups. ^**^*p* < 0.01,^***^*p* < 0.001. Data are presented as mean ± SD.


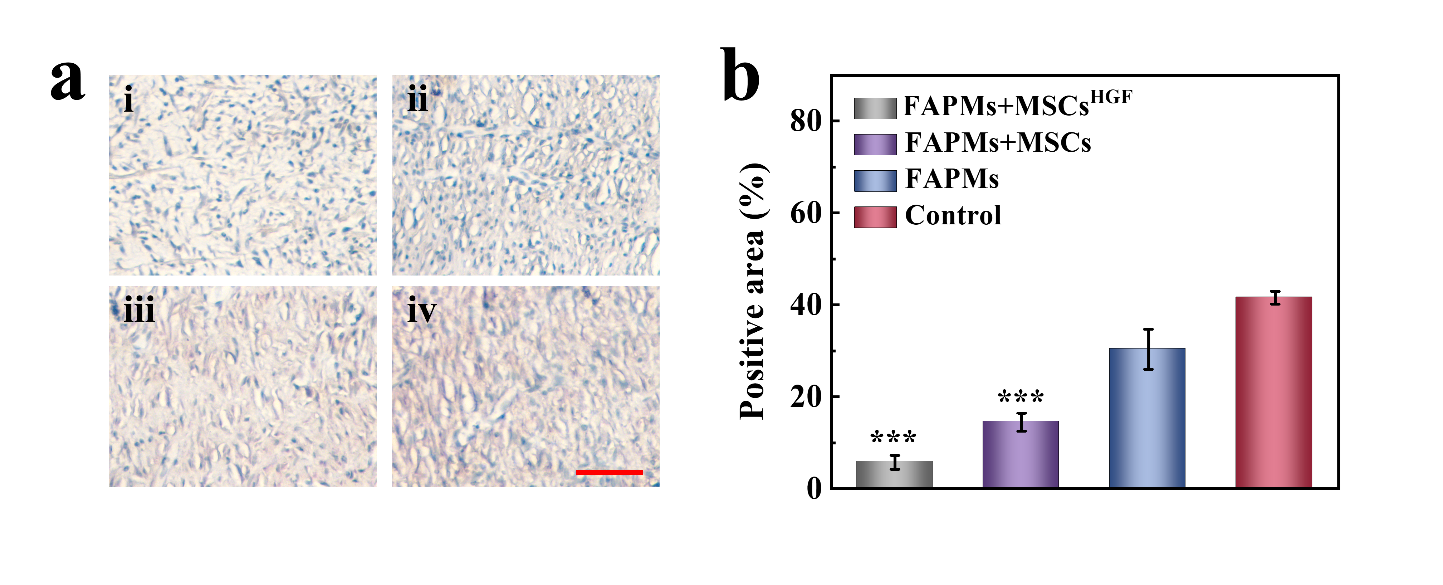


**Figure. S10 | Immunostaining of TNF-α of granulation tissue in different groups. (a)** Microscope photographs. **(b)** Statistical analysis (n=3). i) the FAPMs+MSCs^HGF^ group, ii) the FAPMs+MSC group, iii) the FAPMs groups, and iv) the control group. The scale bar is 200 μm.A Student's *t*-test was performed to compare the control group and experimental groups. ^***^*p* < 0.001. Data are presented as mean ± SD.


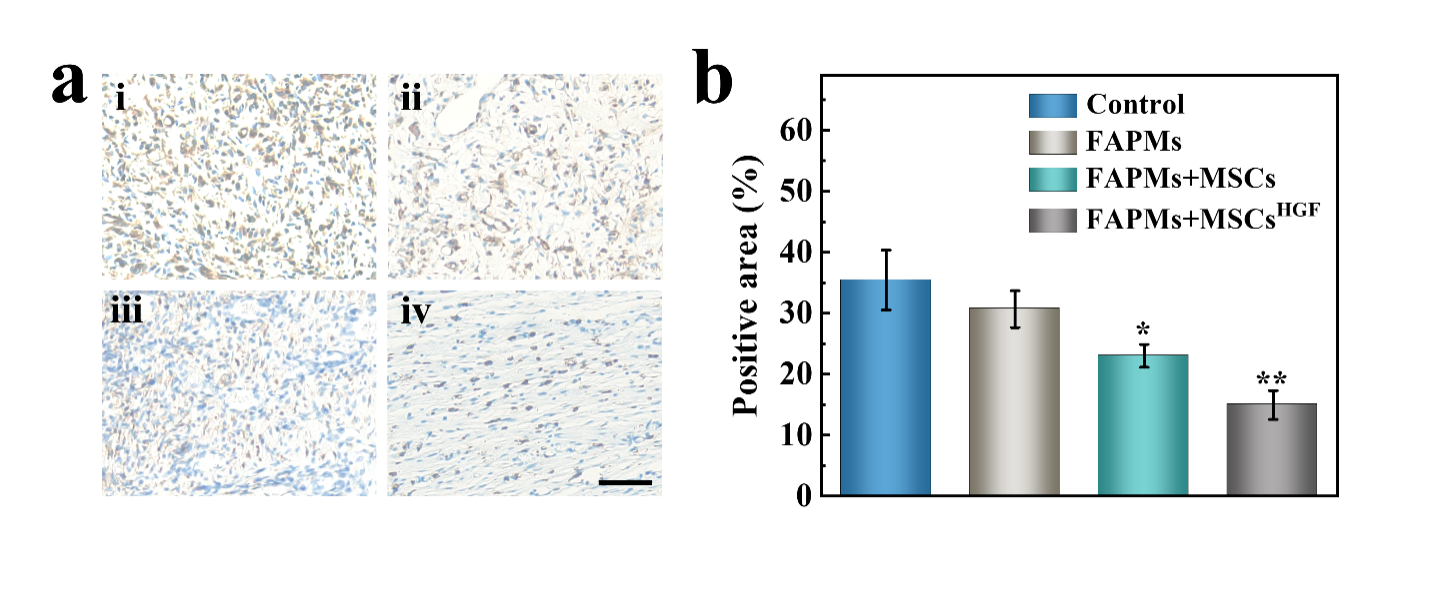


**Figure. S11 | Immunostaining of iNOS of granulation tissue in different groups.** **(a)** Microscope photographs. **(b)** Statistical analysis (n=3). i) the control group, ii) the FAPMs group, iii) the FAPMs+MSCs groups, and iv) the FAPMs+MSCs^HGF^ group. The scale bar is 100 μm. A Student's *t*-test was performed to compare the control group and experimental groups. ^*^*p* < 0.05,^**^*p* < 0.01.Data are presented as mean ± SD.
