# Supplementary figures and images for: Freeze-Derived Anisotropic Porous Microparticles for Engineered Mesenchymal Stem Cell Loading and Wound Healing
Source: Research (Wash D C). 2025 Apr 22;8:0668. doi: 10.34133/research.0668 (PMC12012297; doi:10.34133/research.0668)

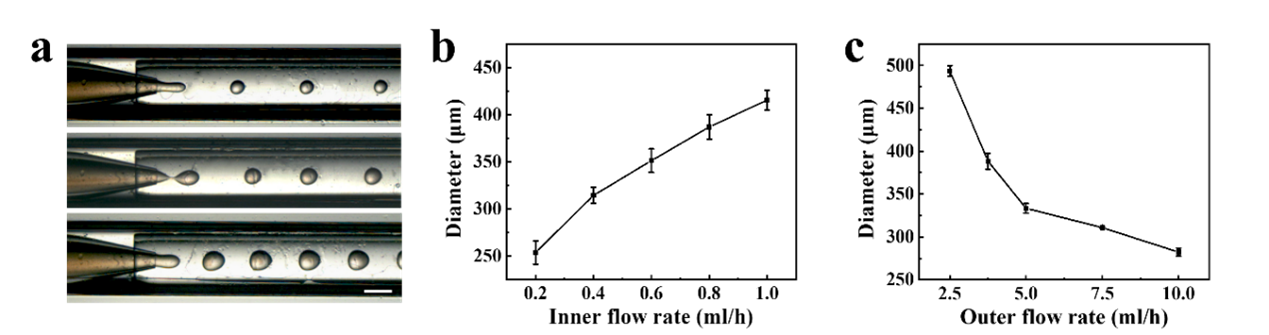

Supplement: Supplementary 1 — Figs. S1 to S11 [file research.0668.f1.zip › Figure S1.tif]

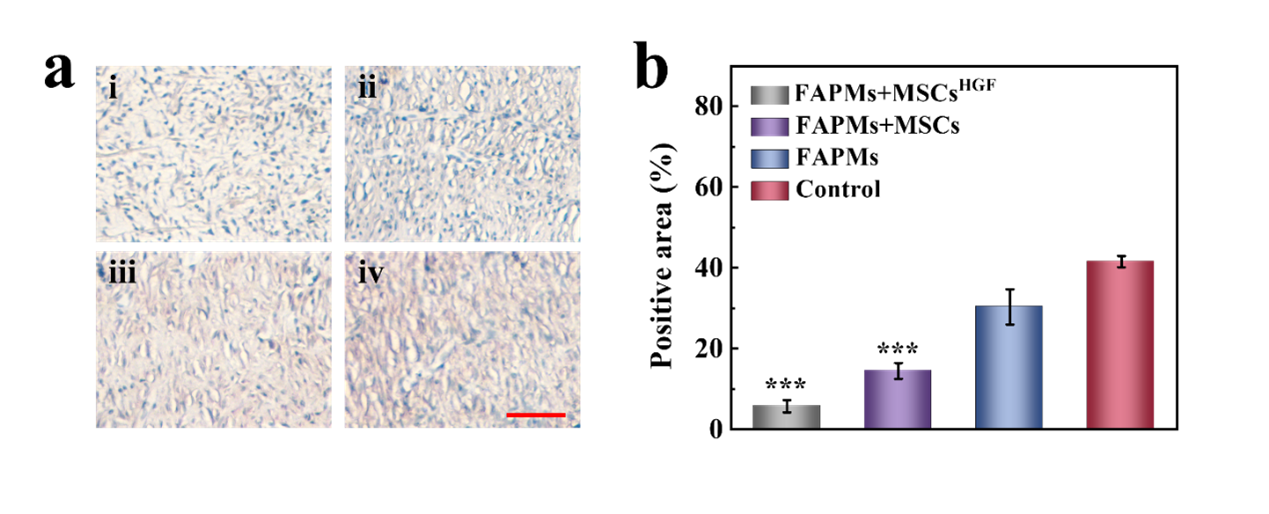

Supplement: Supplementary 1 — Figs. S1 to S11 [file research.0668.f1.zip › Figure S10.tif]

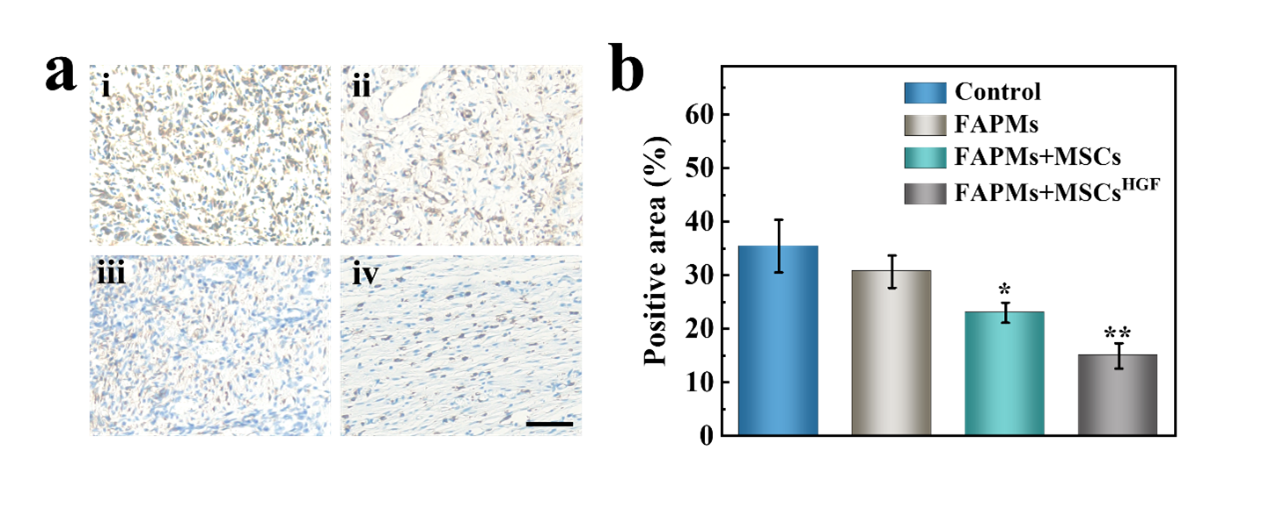

Supplement: Supplementary 1 — Figs. S1 to S11 [file research.0668.f1.zip › Figure S11.tif]

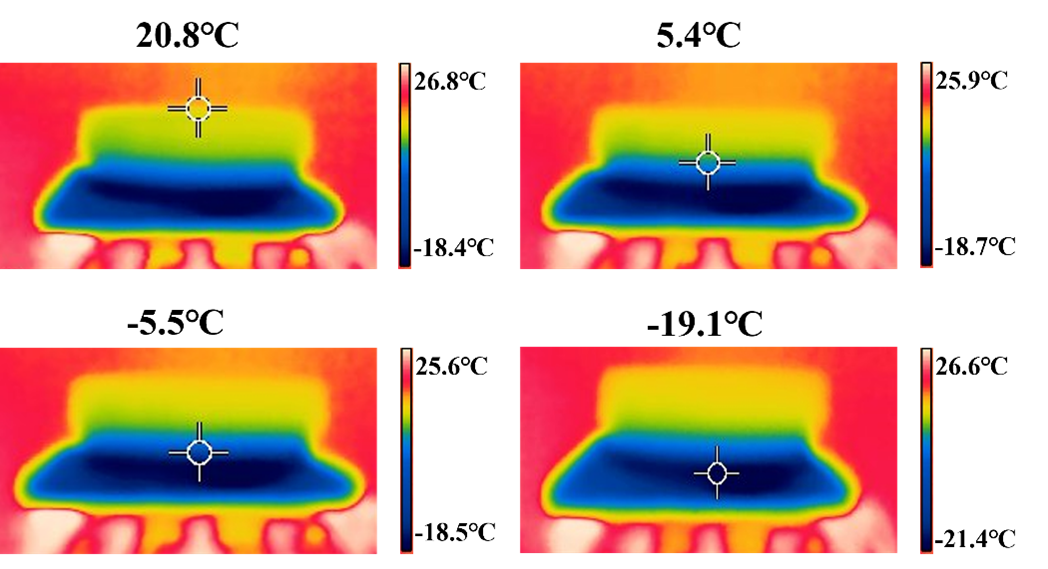

Supplement: Supplementary 1 — Figs. S1 to S11 [file research.0668.f1.zip › Figure S2.tif]

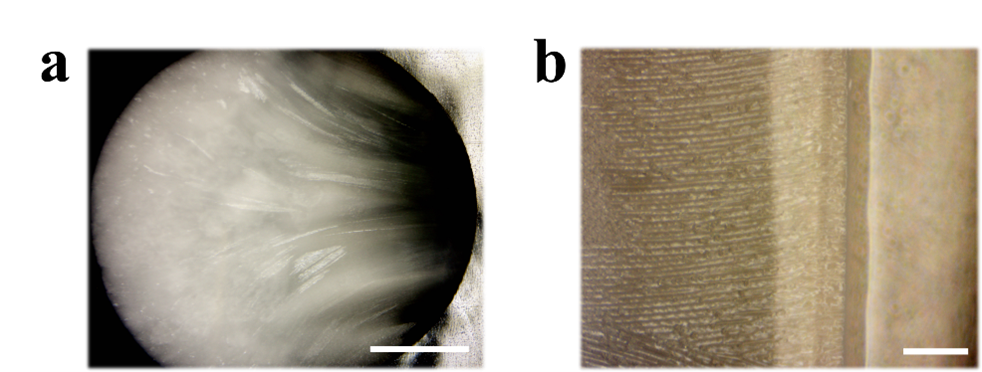

Supplement: Supplementary 1 — Figs. S1 to S11 [file research.0668.f1.zip › Figure S3.tif]

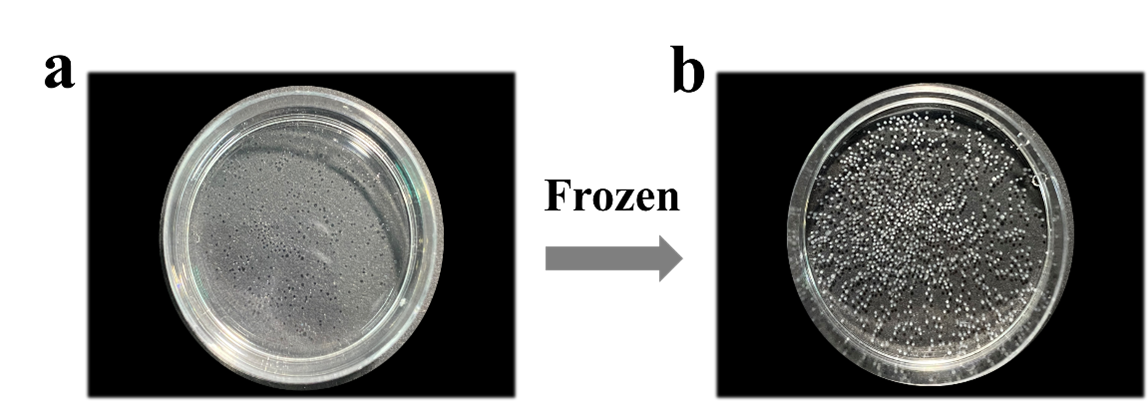

Supplement: Supplementary 1 — Figs. S1 to S11 [file research.0668.f1.zip › Figure S4.tif]

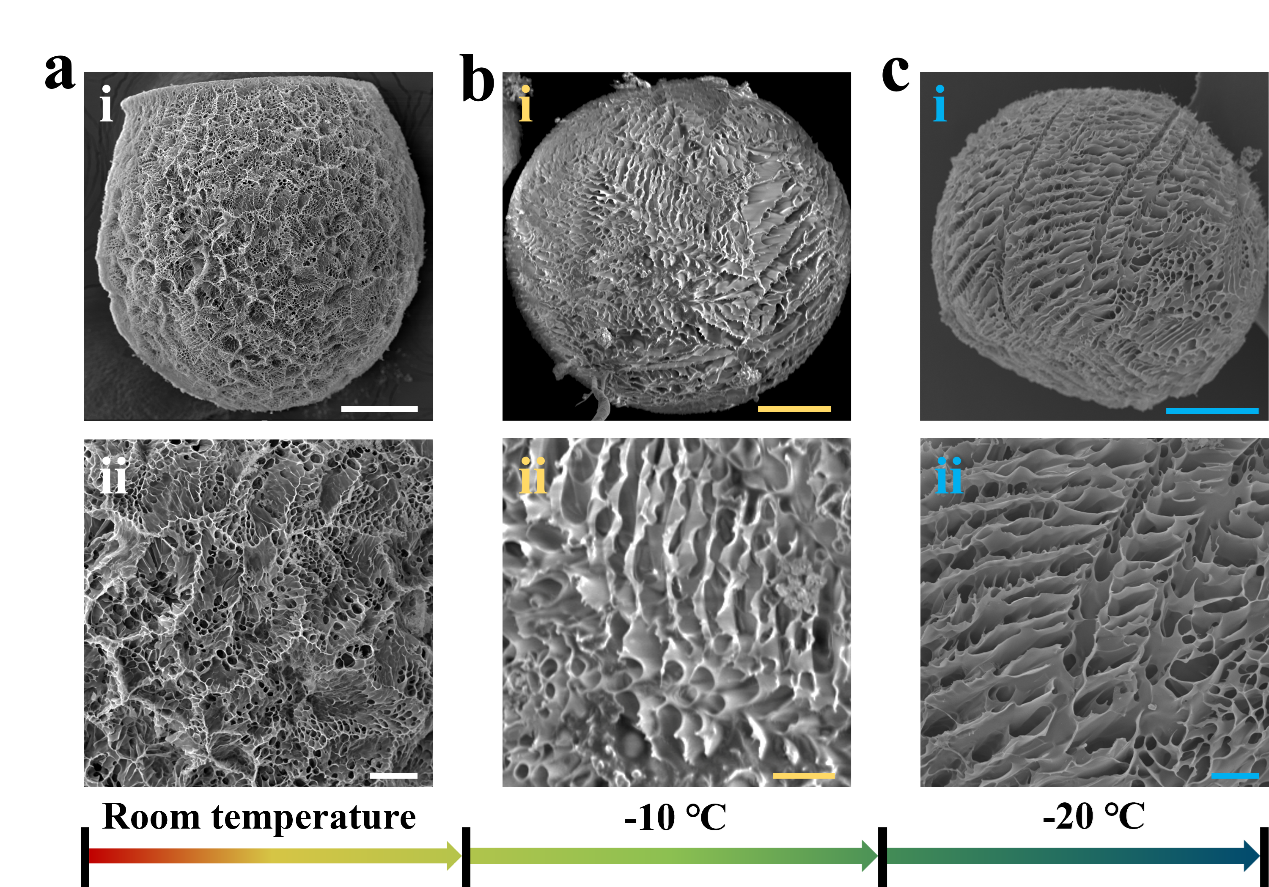

Supplement: Supplementary 1 — Figs. S1 to S11 [file research.0668.f1.zip › Figure S5.tif]

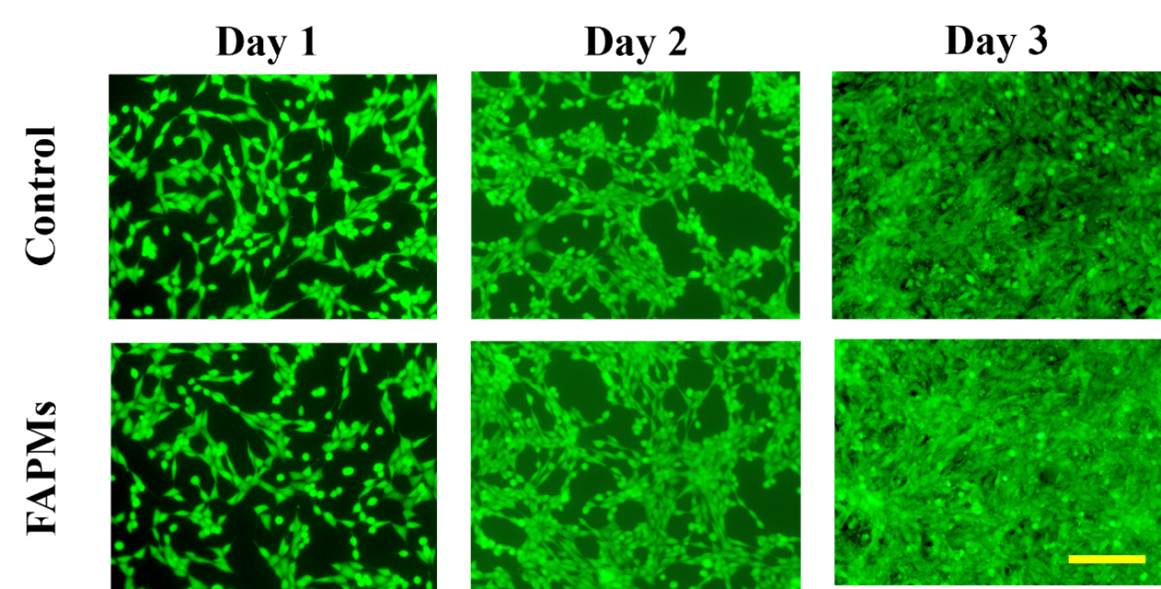

Supplement: Supplementary 1 — Figs. S1 to S11 [file research.0668.f1.zip › Figure S6.tif]

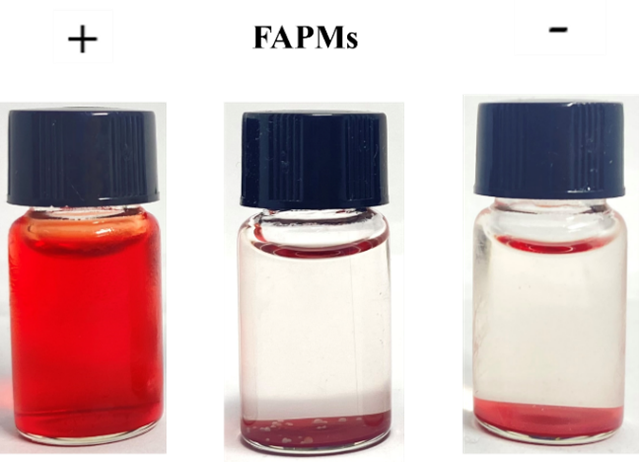

Supplement: Supplementary 1 — Figs. S1 to S11 [file research.0668.f1.zip › Figure S7.tif]

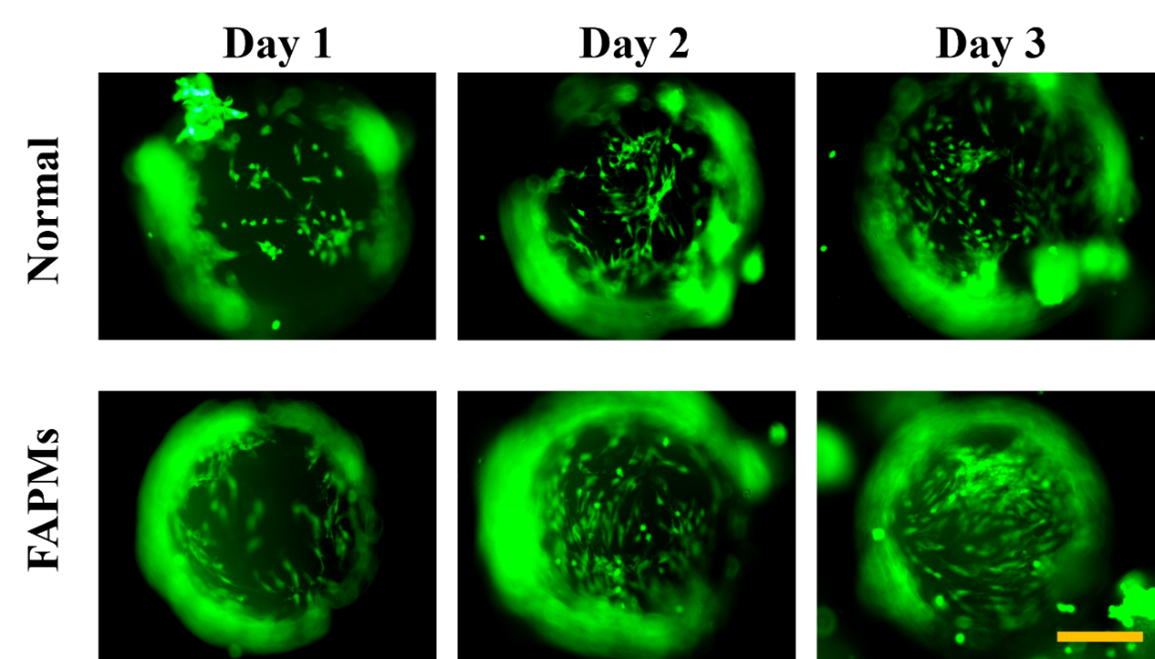

Supplement: Supplementary 1 — Figs. S1 to S11 [file research.0668.f1.zip › Figure S8.tif]

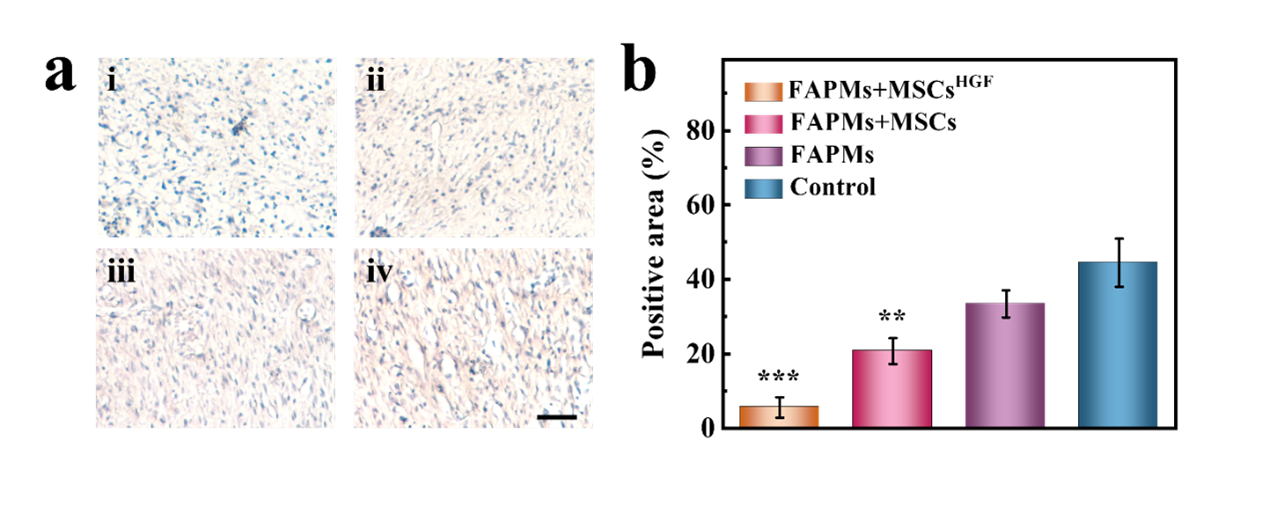

Supplement: Supplementary 1 — Figs. S1 to S11 [file research.0668.f1.zip › Figure S9.tif]
